# 1-(12-Azido-*n*-dodec­yl)-4-[(1,5-dibenzyl-2,4-dioxo-2,3,4,5-tetra­hydro-1*H*-1,5-benzodiazepin-3-yl)meth­yl]-1*H*-1,2,3-triazole

**DOI:** 10.1107/S1600536809054464

**Published:** 2009-12-24

**Authors:** Hind Jabli, F. Ouazzani Chahdi, Natalie Saffon, El Mokhtar Essassi, Seik Weng Ng

**Affiliations:** a Laboratoire de Chimie Organique Appliquée, Faculté des Sciences et Techniques, Université Sidi Mohamed Ben Abdallah, Fés, Morocco; b Service Commun Rayons-X FR2599, Université Paul Sabatier, Bâtiment 2R1, 118 Route de Narbonne, Toulouse, France; c Laboratoire de Chimie Organique Hétérocyclique, Pôle de Compétences Pharmacochimie, Université Mohammed V-Agdal, BP 1014 Avenue Ibn Batout, Rabat, Morocco; d Department of Chemistry, University of Malaya, 50603 Kuala Lumpur, Malaysia

## Abstract

The reaction of 1,5-dibenzyl-3-propargyl-1,5-benzodiazepine-2,4-dione with 1,12-diazido-*n*-dodecane in the presence of catalysts leads to the formation of the title compound, C_38_H_46_N_8_O_2_. The seven-membered diazepinyl ring adopts a boat conformation with the azido­dodecyl­triazolylmethyl-bearing C atom as the prow and the fused-ring C atoms as the stern. The octyltriazolylmethyl substituent occupies an axial position.

## Related literature

For the crystal structures of other *N*-substituted homologs, see: Jabli *et al.* (2009 ▶, 2010 ▶).
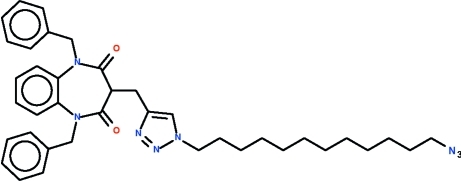


## Experimental

### 

#### Crystal data

C_38_H_46_N_8_O_2_
*M* *_r_* = 646.83
Triclinic, 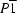

*a* = 9.4269 (2) Å
*b* = 12.3304 (3) Å
*c* = 15.5500 (4) Å
α = 101.121 (2)°
β = 92.175 (2)°
γ = 92.935 (2)°
*V* = 1769.11 (7) Å^3^
*Z* = 2
Mo *K*α radiation
μ = 0.08 mm^−1^
*T* = 193 K
0.40 × 0.20 × 0.06 mm

#### Data collection

Bruker APEXII diffractometer
33681 measured reflections
8126 independent reflections
4315 reflections with *I* > 2σ(*I*)
*R*_int_ = 0.076

#### Refinement

*R*[*F* ^2^ > 2σ(*F* ^2^)] = 0.052
*wR*(*F* ^2^) = 0.147
*S* = 1.00
8126 reflections
433 parameters
H-atom parameters constrained
Δρ_max_ = 0.21 e Å^−3^
Δρ_min_ = −0.22 e Å^−3^

### 

Data collection: *APEX2* (Bruker, 2005 ▶); cell refinement: *SAINT* (Bruker, 2005 ▶); data reduction: *SAINT*; program(s) used to solve structure: *SHELXS97* (Sheldrick, 2008 ▶); program(s) used to refine structure: *SHELXL97* (Sheldrick, 2008 ▶); molecular graphics: *X-SEED* (Barbour, 2001 ▶); software used to prepare material for publication: *publCIF* (Westrip, 2010 ▶).

## Supplementary Material

Crystal structure: contains datablocks global, I. DOI: 10.1107/S1600536809054464/bt5146sup1.cif

Structure factors: contains datablocks I. DOI: 10.1107/S1600536809054464/bt5146Isup2.hkl

Additional supplementary materials:  crystallographic information; 3D view; checkCIF report

## Figures and Tables

**Fig. 1.**
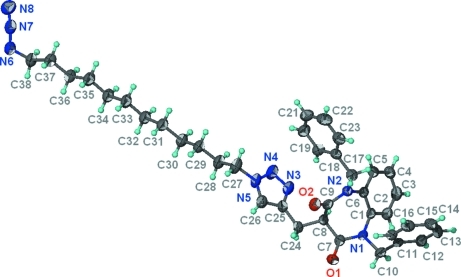
Thermal ellipsoid plot (Barbour, 2001) of C38H46N8O2 at the 70% probability level; hydrogen atoms are drawn as arbitrary radius.

## supplementary crystallographic information

### Experimental

To a solution 1,5-dibenzyl-3-propargyl-1,5-benzodiazepine-2,4-dione (1 mmol) *t*-butyl alcohol/water (1/2, 8 ml) was added copper sulfate pentahydrate (1 mmol), sodium ascorbate (2 mmol) and 1,12-diazido-*n*-dodecaneoctane (5 mmol). Stirring was continued for 12 h. The solution was diluted with water (20 ml) and the organic compound extracted with ethyl acetate (2 *x* 20 ml). The extracts were washed with brine and dried over sodium sulfate. The compound was recrystallized from ethyl acetate/ether to give colorless crystals.

### Refinement

Carbon-bound H-atoms were placed in calculated positions (C—H 0.95 to 0.99 Å) and were included in the refinement in the riding model approximation, with *U*(H) set to 1.2–1.5*U*(C).

### Crystal data

**Table:**

C_38_H_46_N_8_O_2_	*Z* = 2
*M**_r_* = 646.83	*F*(000) = 692
Triclinic, *P*1	*D*_x_ = 1.214 Mg m^−^^3^
Hall symbol: -P 1	Mo *K*α radiation, λ = 0.71073 Å
*a* = 9.4269 (2) Å	Cell parameters from 3120 reflections
*b* = 12.3304 (3) Å	θ = 2.3–22.9°
*c* = 15.5500 (4) Å	µ = 0.08 mm^−^^1^
α = 101.121 (2)°	*T* = 193 K
β = 92.175 (2)°	Plate, colorless
γ = 92.935 (2)°	0.40 × 0.20 × 0.06 mm
*V* = 1769.11 (7) Å^3^	

### Data collection

**Table:**

Bruker APEXII diffractometer	4315 reflections with *I* > 2σ(*I*)
Radiation source: fine-focus sealed tube	*R*_int_ = 0.076
graphite	θ_max_ = 27.5°, θ_min_ = 2.2°
φ and ω scans	*h* = −12→12
33681 measured reflections	*k* = −16→16
8126 independent reflections	*l* = −20→20

### Refinement

**Table:**

Refinement on *F*^2^	Primary atom site location: structure-invariant direct methods
Least-squares matrix: full	Secondary atom site location: difference Fourier map
*R*[*F*^2^ > 2σ(*F*^2^)] = 0.052	Hydrogen site location: inferred from neighbouring sites
*wR*(*F*^2^) = 0.147	H-atom parameters constrained
*S* = 1.00	*w* = 1/[σ^2^(*F*_o_^2^) + (0.0637*P*)^2^ + 0.0087*P*] where *P* = (*F*_o_^2^ + 2*F*_c_^2^)/3
8126 reflections	(Δ/σ)_max_ = 0.001
433 parameters	Δρ_max_ = 0.21 e Å^−^^3^
0 restraints	Δρ_min_ = −0.22 e Å^−^^3^

### Fractional atomic coordinates and isotropic or equivalent isotropic displacement parameters (Å^2^)

**Table:**

	*x*	*y*	*z*	*U*_iso_*/*U*_eq_	
O1	0.49525 (16)	1.12343 (12)	0.23925 (10)	0.0408 (4)	
O2	0.51737 (15)	0.90533 (11)	0.37285 (9)	0.0367 (4)	
N1	0.27355 (18)	1.11470 (12)	0.29224 (11)	0.0297 (4)	
N2	0.28200 (18)	0.93241 (13)	0.38543 (11)	0.0290 (4)	
N3	0.3838 (2)	0.70440 (15)	0.17514 (14)	0.0477 (5)	
N4	0.4145 (2)	0.60090 (15)	0.17564 (15)	0.0535 (6)	
N5	0.5564 (2)	0.60077 (14)	0.18490 (13)	0.0429 (5)	
N6	1.0314 (2)	−0.65890 (16)	−0.19426 (15)	0.0516 (6)	
N7	0.9694 (2)	−0.73485 (17)	−0.16605 (13)	0.0433 (5)	
N8	0.9271 (2)	−0.81025 (18)	−0.14108 (15)	0.0553 (6)	
C1	0.1478 (2)	1.05032 (15)	0.30484 (13)	0.0283 (5)	
C2	0.0170 (2)	1.07803 (17)	0.27332 (14)	0.0353 (5)	
H2	0.0139	1.1362	0.2413	0.042*	
C3	−0.1078 (2)	1.02300 (18)	0.28764 (15)	0.0405 (6)	
H3	−0.1960	1.0432	0.2657	0.049*	
C4	−0.1046 (2)	0.93793 (18)	0.33418 (15)	0.0377 (5)	
H4	−0.1906	0.9003	0.3449	0.045*	
C5	0.0246 (2)	0.90793 (17)	0.36500 (14)	0.0340 (5)	
H5	0.0267	0.8490	0.3963	0.041*	
C6	0.1516 (2)	0.96343 (15)	0.35062 (13)	0.0278 (5)	
C7	0.3922 (2)	1.06848 (16)	0.25614 (13)	0.0297 (5)	
C8	0.3893 (2)	0.94297 (15)	0.24558 (13)	0.0271 (5)	
H8	0.2952	0.9099	0.2183	0.033*	
C9	0.4037 (2)	0.92453 (15)	0.33936 (13)	0.0288 (5)	
C10	0.2720 (2)	1.23642 (15)	0.31792 (14)	0.0345 (5)	
H10A	0.2054	1.2641	0.2775	0.041*	
H10B	0.3682	1.2700	0.3126	0.041*	
C11	0.2275 (2)	1.27102 (15)	0.41053 (14)	0.0286 (5)	
C12	0.1029 (2)	1.32471 (17)	0.42944 (15)	0.0364 (5)	
H12	0.0435	1.3395	0.3828	0.044*	
C13	0.0643 (3)	1.35682 (18)	0.51513 (17)	0.0452 (6)	
H13	−0.0212	1.3933	0.5271	0.054*	
C14	0.1498 (3)	1.33594 (18)	0.58312 (16)	0.0459 (6)	
H14	0.1236	1.3584	0.6420	0.055*	
C15	0.2740 (3)	1.28227 (18)	0.56568 (15)	0.0415 (6)	
H15	0.3332	1.2678	0.6125	0.050*	
C16	0.3115 (2)	1.24989 (16)	0.48007 (14)	0.0343 (5)	
H16	0.3964	1.2124	0.4684	0.041*	
C17	0.2891 (2)	0.91041 (17)	0.47532 (13)	0.0333 (5)	
H17A	0.2030	0.9374	0.5050	0.040*	
H17B	0.3723	0.9538	0.5079	0.040*	
C18	0.3006 (2)	0.78970 (17)	0.48126 (14)	0.0310 (5)	
C19	0.3054 (2)	0.70492 (17)	0.40862 (15)	0.0379 (5)	
H19	0.2973	0.7207	0.3513	0.046*	
C20	0.3221 (2)	0.59694 (18)	0.41938 (17)	0.0451 (6)	
H20	0.3264	0.5398	0.3691	0.054*	
C21	0.3323 (2)	0.5715 (2)	0.50144 (19)	0.0496 (7)	
H21	0.3434	0.4974	0.5082	0.060*	
C22	0.3262 (3)	0.6553 (2)	0.57389 (18)	0.0522 (7)	
H22	0.3330	0.6387	0.6310	0.063*	
C23	0.3104 (2)	0.76358 (19)	0.56407 (15)	0.0415 (6)	
H23	0.3061	0.8204	0.6146	0.050*	
C24	0.5083 (2)	0.89162 (16)	0.19044 (14)	0.0316 (5)	
H24A	0.4979	0.9081	0.1308	0.038*	
H24B	0.6012	0.9255	0.2170	0.038*	
C25	0.5065 (2)	0.76899 (17)	0.18398 (14)	0.0328 (5)	
C26	0.6166 (2)	0.70312 (17)	0.19058 (15)	0.0387 (6)	
H26	0.7151	0.7250	0.1977	0.046*	
C27	0.6233 (3)	0.49812 (18)	0.19414 (17)	0.0509 (7)	
H27A	0.5770	0.4673	0.2410	0.061*	
H27B	0.7246	0.5163	0.2129	0.061*	
C28	0.6142 (3)	0.41098 (18)	0.11141 (16)	0.0466 (6)	
H28A	0.6623	0.4401	0.0644	0.056*	
H28B	0.5132	0.3920	0.0920	0.056*	
C29	0.6850 (3)	0.30673 (17)	0.12757 (16)	0.0422 (6)	
H29A	0.7868	0.3262	0.1442	0.051*	
H29B	0.6407	0.2817	0.1777	0.051*	
C30	0.6733 (3)	0.21180 (17)	0.04916 (16)	0.0454 (6)	
H30A	0.7185	0.2360	−0.0009	0.054*	
H30B	0.5716	0.1921	0.0322	0.054*	
C31	0.7439 (3)	0.10941 (17)	0.06794 (15)	0.0427 (6)	
H31A	0.8459	0.1293	0.0839	0.051*	
H31B	0.7003	0.0870	0.1193	0.051*	
C32	0.7317 (3)	0.01086 (17)	−0.00845 (15)	0.0430 (6)	
H32A	0.6299	−0.0072	−0.0260	0.052*	
H32B	0.7789	0.0322	−0.0590	0.052*	
C33	0.7970 (3)	−0.09223 (17)	0.01205 (15)	0.0430 (6)	
H33A	0.8974	−0.0733	0.0330	0.052*	
H33B	0.7457	−0.1167	0.0600	0.052*	
C34	0.7915 (3)	−0.18720 (17)	−0.06687 (15)	0.0429 (6)	
H34A	0.6912	−0.2044	−0.0883	0.051*	
H34B	0.8438	−0.1624	−0.1143	0.051*	
C35	0.8535 (3)	−0.29282 (17)	−0.04934 (15)	0.0455 (6)	
H35A	0.7937	−0.3236	−0.0077	0.055*	
H35B	0.9499	−0.2745	−0.0212	0.055*	
C36	0.8626 (3)	−0.38058 (17)	−0.13207 (15)	0.0397 (6)	
H36A	0.9195	−0.3486	−0.1743	0.048*	
H36B	0.7656	−0.4001	−0.1591	0.048*	
C37	0.9285 (3)	−0.48615 (17)	−0.11688 (15)	0.0396 (6)	
H37A	1.0227	−0.4671	−0.0858	0.048*	
H37C	0.8671	−0.5229	−0.0794	0.048*	
C38	0.9452 (3)	−0.56462 (18)	−0.20306 (15)	0.0432 (6)	
H38C	0.8497	−0.5935	−0.2287	0.052*	
H38A	0.9903	−0.5230	−0.2443	0.052*	

### Atomic displacement parameters (Å^2^)

**Table:**

	*U*^11^	*U*^22^	*U*^33^	*U*^12^	*U*^13^	*U*^23^
O1	0.0375 (10)	0.0340 (8)	0.0512 (10)	−0.0034 (7)	0.0123 (8)	0.0086 (7)
O2	0.0314 (9)	0.0370 (8)	0.0403 (9)	0.0042 (7)	−0.0061 (7)	0.0049 (7)
N1	0.0317 (10)	0.0210 (8)	0.0362 (11)	0.0022 (7)	0.0042 (8)	0.0046 (7)
N2	0.0294 (10)	0.0297 (9)	0.0279 (10)	0.0015 (7)	0.0025 (8)	0.0058 (7)
N3	0.0338 (12)	0.0308 (11)	0.0760 (16)	0.0051 (9)	0.0020 (10)	0.0035 (10)
N4	0.0373 (13)	0.0324 (11)	0.0869 (17)	0.0053 (9)	−0.0011 (11)	0.0018 (10)
N5	0.0375 (12)	0.0319 (10)	0.0561 (13)	0.0098 (9)	−0.0019 (10)	−0.0004 (9)
N6	0.0459 (13)	0.0370 (12)	0.0724 (16)	0.0090 (10)	0.0151 (11)	0.0079 (11)
N7	0.0390 (13)	0.0407 (12)	0.0462 (13)	0.0067 (10)	−0.0021 (10)	−0.0016 (10)
N8	0.0532 (15)	0.0488 (13)	0.0639 (15)	0.0019 (11)	−0.0071 (11)	0.0134 (11)
C1	0.0290 (12)	0.0226 (10)	0.0312 (12)	0.0031 (8)	0.0027 (9)	−0.0007 (8)
C2	0.0334 (13)	0.0345 (12)	0.0388 (13)	0.0066 (10)	−0.0002 (10)	0.0082 (10)
C3	0.0308 (14)	0.0428 (13)	0.0454 (15)	0.0072 (10)	−0.0023 (11)	0.0020 (11)
C4	0.0291 (13)	0.0388 (13)	0.0420 (14)	−0.0026 (10)	0.0064 (10)	−0.0002 (10)
C5	0.0356 (14)	0.0301 (11)	0.0347 (13)	−0.0006 (9)	0.0066 (10)	0.0019 (9)
C6	0.0282 (12)	0.0244 (10)	0.0293 (12)	0.0022 (8)	0.0020 (9)	0.0010 (8)
C7	0.0319 (13)	0.0284 (11)	0.0284 (12)	0.0036 (9)	−0.0002 (9)	0.0043 (9)
C8	0.0236 (11)	0.0269 (10)	0.0301 (12)	0.0023 (8)	0.0027 (9)	0.0030 (9)
C9	0.0302 (13)	0.0215 (10)	0.0331 (12)	−0.0002 (8)	−0.0015 (10)	0.0026 (9)
C10	0.0425 (14)	0.0226 (10)	0.0387 (13)	0.0029 (9)	0.0039 (10)	0.0059 (9)
C11	0.0291 (12)	0.0207 (10)	0.0367 (13)	−0.0002 (8)	0.0043 (9)	0.0072 (9)
C12	0.0300 (13)	0.0332 (12)	0.0460 (15)	0.0038 (9)	−0.0025 (11)	0.0077 (10)
C13	0.0353 (14)	0.0388 (13)	0.0604 (18)	0.0075 (10)	0.0163 (12)	0.0031 (12)
C14	0.0559 (17)	0.0396 (13)	0.0404 (15)	−0.0048 (12)	0.0168 (13)	0.0029 (11)
C15	0.0476 (16)	0.0424 (13)	0.0364 (14)	−0.0009 (11)	0.0015 (11)	0.0131 (11)
C16	0.0323 (13)	0.0308 (11)	0.0419 (14)	0.0044 (9)	0.0057 (10)	0.0106 (10)
C17	0.0354 (13)	0.0372 (12)	0.0265 (12)	0.0018 (9)	0.0000 (9)	0.0045 (9)
C18	0.0228 (12)	0.0360 (12)	0.0357 (13)	0.0000 (9)	−0.0001 (9)	0.0117 (10)
C19	0.0408 (14)	0.0341 (12)	0.0395 (14)	0.0008 (10)	−0.0002 (11)	0.0093 (10)
C20	0.0427 (15)	0.0311 (12)	0.0605 (17)	0.0018 (10)	0.0032 (12)	0.0067 (11)
C21	0.0360 (15)	0.0412 (14)	0.078 (2)	0.0028 (11)	0.0024 (13)	0.0271 (14)
C22	0.0406 (16)	0.0661 (18)	0.0603 (18)	0.0024 (13)	−0.0007 (13)	0.0395 (15)
C23	0.0370 (14)	0.0509 (14)	0.0382 (14)	0.0025 (11)	0.0023 (11)	0.0128 (11)
C24	0.0283 (12)	0.0338 (11)	0.0325 (12)	0.0041 (9)	0.0039 (9)	0.0046 (9)
C25	0.0307 (13)	0.0334 (12)	0.0330 (12)	0.0048 (9)	0.0039 (10)	0.0018 (9)
C26	0.0319 (13)	0.0351 (13)	0.0467 (14)	0.0046 (10)	0.0015 (11)	0.0016 (10)
C27	0.0567 (18)	0.0363 (13)	0.0586 (17)	0.0159 (12)	−0.0042 (13)	0.0041 (12)
C28	0.0526 (17)	0.0350 (13)	0.0517 (16)	0.0109 (11)	0.0081 (12)	0.0042 (11)
C29	0.0463 (15)	0.0317 (12)	0.0494 (15)	0.0081 (10)	0.0062 (12)	0.0075 (11)
C30	0.0521 (16)	0.0359 (13)	0.0498 (16)	0.0127 (11)	0.0081 (12)	0.0085 (11)
C31	0.0536 (16)	0.0342 (12)	0.0422 (14)	0.0107 (11)	0.0082 (12)	0.0084 (11)
C32	0.0515 (16)	0.0370 (13)	0.0417 (14)	0.0115 (11)	0.0071 (11)	0.0074 (10)
C33	0.0567 (17)	0.0371 (13)	0.0372 (14)	0.0157 (11)	0.0109 (12)	0.0069 (10)
C34	0.0496 (16)	0.0363 (13)	0.0430 (14)	0.0116 (11)	0.0051 (11)	0.0053 (10)
C35	0.0618 (18)	0.0374 (13)	0.0383 (14)	0.0141 (12)	0.0077 (12)	0.0058 (11)
C36	0.0437 (15)	0.0357 (12)	0.0395 (14)	0.0092 (10)	0.0010 (11)	0.0055 (10)
C37	0.0441 (15)	0.0353 (12)	0.0379 (14)	0.0073 (10)	−0.0031 (11)	0.0032 (10)
C38	0.0453 (15)	0.0370 (13)	0.0462 (15)	0.0101 (11)	0.0056 (11)	0.0030 (11)

### Geometric parameters (Å, °)

**Table:**

O1—C7	1.222 (2)	C19—C20	1.389 (3)
O2—C9	1.228 (2)	C19—H19	0.9500
N1—C7	1.371 (3)	C20—C21	1.373 (3)
N1—C1	1.434 (3)	C20—H20	0.9500
N1—C10	1.478 (2)	C21—C22	1.379 (3)
N2—C9	1.373 (3)	C21—H21	0.9500
N2—C6	1.424 (2)	C22—C23	1.388 (3)
N2—C17	1.474 (2)	C22—H22	0.9500
N3—N4	1.324 (2)	C23—H23	0.9500
N3—C25	1.357 (3)	C24—C25	1.495 (3)
N4—N5	1.340 (3)	C24—H24A	0.9900
N5—C26	1.342 (3)	C24—H24B	0.9900
N5—C27	1.471 (3)	C25—C26	1.363 (3)
N6—N7	1.238 (3)	C26—H26	0.9500
N6—C38	1.477 (3)	C27—C28	1.505 (3)
N7—N8	1.135 (3)	C27—H27A	0.9900
C1—C2	1.393 (3)	C27—H27B	0.9900
C1—C6	1.397 (3)	C28—C29	1.536 (3)
C2—C3	1.375 (3)	C28—H28A	0.9900
C2—H2	0.9500	C28—H28B	0.9900
C3—C4	1.385 (3)	C29—C30	1.515 (3)
C3—H3	0.9500	C29—H29A	0.9900
C4—C5	1.385 (3)	C29—H29B	0.9900
C4—H4	0.9500	C30—C31	1.528 (3)
C5—C6	1.395 (3)	C30—H30A	0.9900
C5—H5	0.9500	C30—H30B	0.9900
C7—C8	1.523 (3)	C31—C32	1.523 (3)
C8—C9	1.521 (3)	C31—H31A	0.9900
C8—C24	1.527 (3)	C31—H31B	0.9900
C8—H8	1.0000	C32—C33	1.523 (3)
C10—C11	1.503 (3)	C32—H32A	0.9900
C10—H10A	0.9900	C32—H32B	0.9900
C10—H10B	0.9900	C33—C34	1.521 (3)
C11—C16	1.389 (3)	C33—H33A	0.9900
C11—C12	1.392 (3)	C33—H33B	0.9900
C12—C13	1.383 (3)	C34—C35	1.521 (3)
C12—H12	0.9500	C34—H34A	0.9900
C13—C14	1.377 (3)	C34—H34B	0.9900
C13—H13	0.9500	C35—C36	1.521 (3)
C14—C15	1.384 (3)	C35—H35A	0.9900
C14—H14	0.9500	C35—H35B	0.9900
C15—C16	1.379 (3)	C36—C37	1.525 (3)
C15—H15	0.9500	C36—H36A	0.9900
C16—H16	0.9500	C36—H36B	0.9900
C17—C18	1.518 (3)	C37—C38	1.513 (3)
C17—H17A	0.9900	C37—H37A	0.9900
C17—H17B	0.9900	C37—H37C	0.9900
C18—C19	1.387 (3)	C38—H38C	0.9900
C18—C23	1.387 (3)	C38—H38A	0.9900
			
C7—N1—C1	123.00 (16)	C18—C23—C22	120.7 (2)
C7—N1—C10	119.34 (17)	C18—C23—H23	119.7
C1—N1—C10	117.65 (16)	C22—C23—H23	119.7
C9—N2—C6	122.66 (17)	C25—C24—C8	111.90 (17)
C9—N2—C17	118.05 (17)	C25—C24—H24A	109.2
C6—N2—C17	119.26 (17)	C8—C24—H24A	109.2
N4—N3—C25	109.03 (18)	C25—C24—H24B	109.2
N3—N4—N5	106.74 (18)	C8—C24—H24B	109.2
N4—N5—C26	110.86 (17)	H24A—C24—H24B	107.9
N4—N5—C27	119.71 (19)	N3—C25—C26	107.91 (19)
C26—N5—C27	129.2 (2)	N3—C25—C24	122.42 (18)
N7—N6—C38	115.6 (2)	C26—C25—C24	129.6 (2)
N8—N7—N6	172.0 (2)	N5—C26—C25	105.5 (2)
C2—C1—C6	118.94 (19)	N5—C26—H26	127.3
C2—C1—N1	118.89 (18)	C25—C26—H26	127.3
C6—C1—N1	122.12 (18)	N5—C27—C28	113.9 (2)
C3—C2—C1	121.4 (2)	N5—C27—H27A	108.8
C3—C2—H2	119.3	C28—C27—H27A	108.8
C1—C2—H2	119.3	N5—C27—H27B	108.8
C2—C3—C4	119.8 (2)	C28—C27—H27B	108.8
C2—C3—H3	120.1	H27A—C27—H27B	107.7
C4—C3—H3	120.1	C27—C28—C29	110.2 (2)
C5—C4—C3	119.8 (2)	C27—C28—H28A	109.6
C5—C4—H4	120.1	C29—C28—H28A	109.6
C3—C4—H4	120.1	C27—C28—H28B	109.6
C4—C5—C6	120.7 (2)	C29—C28—H28B	109.6
C4—C5—H5	119.7	H28A—C28—H28B	108.1
C6—C5—H5	119.7	C30—C29—C28	113.9 (2)
C5—C6—C1	119.40 (19)	C30—C29—H29A	108.8
C5—C6—N2	119.14 (18)	C28—C29—H29A	108.8
C1—C6—N2	121.43 (18)	C30—C29—H29B	108.8
O1—C7—N1	123.06 (18)	C28—C29—H29B	108.8
O1—C7—C8	122.69 (19)	H29A—C29—H29B	107.7
N1—C7—C8	114.09 (17)	C29—C30—C31	112.4 (2)
C9—C8—C7	103.72 (15)	C29—C30—H30A	109.1
C9—C8—C24	111.59 (16)	C31—C30—H30A	109.1
C7—C8—C24	112.98 (17)	C29—C30—H30B	109.1
C9—C8—H8	109.5	C31—C30—H30B	109.1
C7—C8—H8	109.5	H30A—C30—H30B	107.9
C24—C8—H8	109.5	C32—C31—C30	114.22 (19)
O2—C9—N2	121.56 (19)	C32—C31—H31A	108.7
O2—C9—C8	122.38 (19)	C30—C31—H31A	108.7
N2—C9—C8	116.06 (17)	C32—C31—H31B	108.7
N1—C10—C11	111.42 (16)	C30—C31—H31B	108.7
N1—C10—H10A	109.3	H31A—C31—H31B	107.6
C11—C10—H10A	109.3	C33—C32—C31	113.88 (19)
N1—C10—H10B	109.3	C33—C32—H32A	108.8
C11—C10—H10B	109.3	C31—C32—H32A	108.8
H10A—C10—H10B	108.0	C33—C32—H32B	108.8
C16—C11—C12	118.1 (2)	C31—C32—H32B	108.8
C16—C11—C10	120.15 (19)	H32A—C32—H32B	107.7
C12—C11—C10	121.70 (19)	C34—C33—C32	112.85 (19)
C13—C12—C11	120.9 (2)	C34—C33—H33A	109.0
C13—C12—H12	119.6	C32—C33—H33A	109.0
C11—C12—H12	119.6	C34—C33—H33B	109.0
C14—C13—C12	120.0 (2)	C32—C33—H33B	109.0
C14—C13—H13	120.0	H33A—C33—H33B	107.8
C12—C13—H13	120.0	C35—C34—C33	114.86 (19)
C13—C14—C15	120.0 (2)	C35—C34—H34A	108.6
C13—C14—H14	120.0	C33—C34—H34A	108.6
C15—C14—H14	120.0	C35—C34—H34B	108.6
C16—C15—C14	119.7 (2)	C33—C34—H34B	108.6
C16—C15—H15	120.1	H34A—C34—H34B	107.5
C14—C15—H15	120.1	C34—C35—C36	113.11 (19)
C15—C16—C11	121.2 (2)	C34—C35—H35A	109.0
C15—C16—H16	119.4	C36—C35—H35A	109.0
C11—C16—H16	119.4	C34—C35—H35B	109.0
N2—C17—C18	115.03 (17)	C36—C35—H35B	109.0
N2—C17—H17A	108.5	H35A—C35—H35B	107.8
C18—C17—H17A	108.5	C35—C36—C37	114.29 (18)
N2—C17—H17B	108.5	C35—C36—H36A	108.7
C18—C17—H17B	108.5	C37—C36—H36A	108.7
H17A—C17—H17B	107.5	C35—C36—H36B	108.7
C19—C18—C23	118.4 (2)	C37—C36—H36B	108.7
C19—C18—C17	123.59 (19)	H36A—C36—H36B	107.6
C23—C18—C17	117.94 (19)	C38—C37—C36	110.81 (18)
C18—C19—C20	120.3 (2)	C38—C37—H37A	109.5
C18—C19—H19	119.9	C36—C37—H37A	109.5
C20—C19—H19	119.9	C38—C37—H37C	109.5
C21—C20—C19	121.1 (2)	C36—C37—H37C	109.5
C21—C20—H20	119.5	H37A—C37—H37C	108.1
C19—C20—H20	119.5	N6—C38—C37	113.33 (19)
C20—C21—C22	118.9 (2)	N6—C38—H38C	108.9
C20—C21—H21	120.6	C37—C38—H38C	108.9
C22—C21—H21	120.6	N6—C38—H38A	108.9
C21—C22—C23	120.6 (2)	C37—C38—H38A	108.9
C21—C22—H22	119.7	H38C—C38—H38A	107.7
C23—C22—H22	119.7		
			
C25—N3—N4—N5	0.1 (3)	C10—C11—C12—C13	−179.42 (19)
N3—N4—N5—C26	0.2 (3)	C11—C12—C13—C14	0.1 (3)
N3—N4—N5—C27	175.6 (2)	C12—C13—C14—C15	−0.4 (3)
C7—N1—C1—C2	−128.6 (2)	C13—C14—C15—C16	0.0 (3)
C10—N1—C1—C2	50.0 (2)	C14—C15—C16—C11	0.6 (3)
C7—N1—C1—C6	53.9 (3)	C12—C11—C16—C15	−0.8 (3)
C10—N1—C1—C6	−127.5 (2)	C10—C11—C16—C15	179.04 (19)
C6—C1—C2—C3	1.1 (3)	C9—N2—C17—C18	−72.5 (2)
N1—C1—C2—C3	−176.48 (19)	C6—N2—C17—C18	109.0 (2)
C1—C2—C3—C4	−0.1 (3)	N2—C17—C18—C19	0.5 (3)
C2—C3—C4—C5	−0.8 (3)	N2—C17—C18—C23	178.86 (18)
C3—C4—C5—C6	0.8 (3)	C23—C18—C19—C20	−1.1 (3)
C4—C5—C6—C1	0.2 (3)	C17—C18—C19—C20	177.3 (2)
C4—C5—C6—N2	178.23 (18)	C18—C19—C20—C21	0.8 (3)
C2—C1—C6—C5	−1.1 (3)	C19—C20—C21—C22	−0.2 (4)
N1—C1—C6—C5	176.37 (18)	C20—C21—C22—C23	−0.2 (4)
C2—C1—C6—N2	−179.11 (18)	C19—C18—C23—C22	0.8 (3)
N1—C1—C6—N2	−1.6 (3)	C17—C18—C23—C22	−177.7 (2)
C9—N2—C6—C5	138.91 (19)	C21—C22—C23—C18	−0.1 (4)
C17—N2—C6—C5	−42.7 (2)	C9—C8—C24—C25	61.4 (2)
C9—N2—C6—C1	−43.1 (3)	C7—C8—C24—C25	177.80 (17)
C17—N2—C6—C1	135.30 (19)	N4—N3—C25—C26	−0.3 (3)
C1—N1—C7—O1	174.29 (19)	N4—N3—C25—C24	−176.8 (2)
C10—N1—C7—O1	−4.3 (3)	C8—C24—C25—N3	39.0 (3)
C1—N1—C7—C8	−10.3 (3)	C8—C24—C25—C26	−136.7 (2)
C10—N1—C7—C8	171.12 (17)	N4—N5—C26—C25	−0.4 (3)
O1—C7—C8—C9	105.9 (2)	C27—N5—C26—C25	−175.3 (2)
N1—C7—C8—C9	−69.6 (2)	N3—C25—C26—N5	0.4 (2)
O1—C7—C8—C24	−15.1 (3)	C24—C25—C26—N5	176.6 (2)
N1—C7—C8—C24	169.45 (17)	N4—N5—C27—C28	70.5 (3)
C6—N2—C9—O2	174.76 (17)	C26—N5—C27—C28	−115.0 (3)
C17—N2—C9—O2	−3.7 (3)	N5—C27—C28—C29	−179.3 (2)
C6—N2—C9—C8	−4.2 (3)	C27—C28—C29—C30	176.6 (2)
C17—N2—C9—C8	177.39 (16)	C28—C29—C30—C31	−179.6 (2)
C7—C8—C9—O2	−99.5 (2)	C29—C30—C31—C32	178.6 (2)
C24—C8—C9—O2	22.4 (3)	C30—C31—C32—C33	−177.6 (2)
C7—C8—C9—N2	79.4 (2)	C31—C32—C33—C34	−176.5 (2)
C24—C8—C9—N2	−158.65 (16)	C32—C33—C34—C35	−179.1 (2)
C7—N1—C10—C11	−129.1 (2)	C33—C34—C35—C36	−172.9 (2)
C1—N1—C10—C11	52.3 (2)	C34—C35—C36—C37	178.2 (2)
N1—C10—C11—C16	64.5 (2)	C35—C36—C37—C38	−175.1 (2)
N1—C10—C11—C12	−115.7 (2)	N7—N6—C38—C37	78.5 (3)
C16—C11—C12—C13	0.5 (3)	C36—C37—C38—N6	169.6 (2)

## References

[bb1] Barbour, L. J. (2001). *J. Supramol. Chem.* **1**, 189–191.

[bb2] Bruker (2005). *APEX2* and *SAINT* Bruker AXS Inc., Madison, Wisconsin, USA.

[bb3] Jabli, H., Ouazzani Chahdi, F., Garrigues, B., Essassi, E. M. & Ng, S. W. (2009). *Acta Cryst.* E**65**, o3149. 10.1107/S1600536809048508 PMC2971782 21578868

[bb4] Jabli, H., Kandri Rodi, Y., Ladeira, S., Essassi, E. M. & Ng, S. W. (2010). *Acta Cryst.* E**66**, o126. 10.1107/S1600536809052696 PMC2980270 21580016

[bb5] Sheldrick, G. M. (2008). *Acta Cryst.* A**64**, 112–122. 10.1107/S0108767307043930 18156677

[bb6] Westrip, S. P. (2010). *publCIF* In preparation.

